# Reply to the comment on “Accuracy and usability of a diagnostic decision support system in the diagnosis of three representative rheumatic diseases: a randomized controlled trial among medical students”

**DOI:** 10.1186/s13075-022-02806-w

**Published:** 2022-05-24

**Authors:** Johannes Knitza, Axel J. Hueber

**Affiliations:** 1 Department of Internal Medicine 3, Friedrich-Alexander-University Erlangen-Nürnberg (FAU) and Universitätsklinikum Erlangen, Ulmenweg 18, 91054 Erlangen, Germany; 2 Deutsches Zentrum für Immuntherapie (DZI), Friedrich-Alexander-University Erlangen-Nürnberg and Universitätsklinikum Erlangen, Erlangen, Germany; 3 Division of Rheumatology, Klinikum Nürnberg, Paracelsus Medical University, Nürnberg, Germany

With great interest, we read the comment by Gilbert and Wicks on our recent publication [1] testing the accuracy and usability of Ada’s symptom checker among medical students.

We fully agree with Gilbert et al. that the accuracy and usability of the Ada app is extremely user and use-case dependent. Whereas Ada’s accuracy was extremely high (98%) in the rheumatoid arthritis vignette, it was clearly lower for the other two vignettes (granulomatosis with polyangiitis 43%; systemic lupus erythematosus 51%). In a recent publication [2] with actual patients, we analyzed in detail the varying perceived usability regarding Ada compared to a similar rheumatology-specific system (Rheport). Usability of both tools was good, although usability of Ada was significantly lower compared to Rheport. Importantly, usability significantly decreased with age.

The authors of the comment state that the app was designed for layperson users to test what underlying disease might be causing their health issues and state that the app explicitly is not developed for health care professionals (HCP). It is noteworthy that the underlying Ada intelligence is identical to Ada’s HCP focused system. Remarkably, the same authors (Gilbert et al., Ada Health GmbH [3]) published a study in which primary care physicians (GPs) tested 200 clinical vignettes with Ada and other digital symptom assessment apps. Also urgency advice was analyzed. Inspired and analog to that study, we intentionally chose medical students and rheumatology case vignettes (source public online learning center and Rheum2Learn section American college of Rheumatology) with very typical disease symptoms over laypersons to create a “best-case scenario.” Gilbert and Wicks further argue that the used Ada app in our study is not a diagnostic decision support system (DDSS); however, the Ada app provides diagnostic terms upon symptom entry and intentionally recommends urgency advices to support appointments. In our opinion and on the basis of the work by Sutton et al. [4], apps interpreting or translating symptoms into diagnoses fulfill characteristics of DDSS, but could also be named symptom assessment apps, self-diagnosis tools, technology-supported clinical decision support tools, etc. Nevertheless, arguing over the exact description should not distract from proper utilization of this technology, since we believe that DDSS or similar tools could operate through patients or HCPs as a gatekeeper for optimization of patient flows. Unfortunately, these tools still lack accuracy in certain rheumatologic rare diseases.

## Abbreviations

DDSS: Diagnostic decision support system
HCP: Health care professionals
